# Analysis of the influences of social isolation on cognition and the therapeutic potential of deep brain stimulation in a mouse model

**DOI:** 10.3389/fpsyt.2023.1186073

**Published:** 2023-06-20

**Authors:** Yun-Yun Hu, Xuan-Si Ding, Gang Yang, Xue-Song Liang, Lei Feng, Yan-Yun Sun, Rui Chen, Quan-Hong Ma

**Affiliations:** ^1^Department of Neurology and Clinical Research Center of Neurological Disease, The Second Affiliated Hospital of Soochow University, Suzhou, China; ^2^Jiangsu Key Laboratory of Neuropsychiatric Diseases and Institute of Neuroscience, Soochow University, Suzhou, China; ^3^Department of Respiratory Medicine, Sleep Center, The Second Affiliated Hospital of Soochow University, Soochow University, Suzhou, China; ^4^Lab Center, Medical College of Soochow University, Suzhou, China; ^5^Second Clinical College, Dalian Medical University, Dalian, China; ^6^Monash Suzhou Research Institute, Suzhou, China

**Keywords:** social isolation, cognition, emotion, duration-dependent, hypomyelination, deep brain stimulation

## Abstract

**Background:**

Social interaction is a fundamental human need. Social isolation (SI) can have negative effects on both emotional and cognitive function. However, it is currently unclear how age and the duration of SI affect emotion and recognition function. In addition, there is no specific treatment for the effects of SI.

**Methods:**

The adolescence or adult mice were individually housed in cages for 1, 6 or 12 months and for 2 months to estabolish SI mouse model. We investigated the effects of SI on behavior in mice at different ages and under distinct durations of SI, and we explored the possible underlying mechanisms. Then we performed deep brain stimulation (DBS) to evaluate its influences on SI induced behavioral abnormalities.

**Results:**

We found that social recognition was affected in the short term, while social preference was damaged by extremely long periods of SI. In addition to affecting social memory, SI also affects emotion, short-term spatial ability and learning willingness in mice. Myelin was decreased significantly in the medial prefrontal cortex (mPFC) and dorsal hippocampus of socially isolated mice. Cellular activity in response to social stimulation in both areas was impaired by social isolation. By stimulating the mPFC using DBS, we found that DBS alleviated cellular activation disorders in the mPFC after long-term SI and improved social preference in mice.

**Conclusion:**

Our results suggest that the therapeutic potential of stimulating the mPFC with DBS in individuals with social preference deficits caused by long-term social isolation, as well as the effects of DBS on the cellular activity and density of OPCs.

## 1. Introduction

Social interaction is a fundamental human requirement, similar to nutrition and sleep. Social interaction influences our behavior and emotions at different stages of life, regardless of whether it is active or passive. Social isolation (SI), on the other hand, refers to the absence of social contact and communication between an individual and society. SI is a condition in which an individual is isolated from their community and unable to engage in normal social interactions. The ongoing COVID-19 pandemic has resulted in a significant increase in infectious disease control strategies, including quarantine, physical distancing measures, isolation with limited movement and close interaction for people exposed to infectious diseases. This type of social state has raised concerns among researchers about the impact of social isolation on physical and mental health. Previous studies have shown that postnatal social isolation can result in impaired socioemotional development (1, 2), cognitive abilities (3), and motor development (4), while social isolation during adolescence or adulthood may cause depression (5, 6), anxiety (7), reduced sleep and increased mortality (8–10). However, it is not yet known how emotions and behaviors change as the duration of social isolation increases.

The effects of social isolation on brain structure and the mechanisms underlying changes in behaviors have been investigated by various researchers. SI has been shown to decrease the area of the corpus callosum and the volume of some brain regions (11, 12). Myelin, which plays a crucial role in neural signaling and is involved in memory formation and behavior execution, has also been found to be impacted by SI. Some studies have demonstrated that SI affects the content of myelin in specific brain regions and impairs oligodendrogenesis (13–15). Additionally, changes in neuronal activity in specific brain areas can influence social interaction (16, 17). These studies indicate a complex mechanisms involved in interaction between glia and neurons in abnormal behaviors induced by social isolation.

Deep brain stimulation (DBS) is a therapy that delivers current to targeted areas in the brain via electrodes. DBS was initially used for the treatment of movement disorders, such as Parkinson’s disease (PD) (18–20), and its therapeutic efficacy has been established clinically (21, 22). Chronic thalamic stimulation improved tremor and levodopa induced dyskinesias of PD patients (18). Further experiments have shown that DBS can also alleviate memory impairments in PD patients (23). Then DBS exhibits therapeutic effects on patients with memory disorders, such as Rett syndrome (24), Alzheimer’s disease (AD) and dementia (25, 26). In addition, some reports have shown the efficacy of DBS in treating obsessive–compulsive disorder and emotional disorders (27–32). Although DBS has been proven to be an effective physical therapy modality, its ability to rescue changes in behavior and pathology due to social isolation has not yet been studied.

In this study, we socially isolated adolescent and adult mice to investigate the changes in behaviors in mice with different durations of isolation from adolescence and adulthood. We found that changes in behaviors were accompanied by alterations in the content of myelin in specific brain regions. To investigate the potential of DBS in the treatment of the effects of SI, we targeted specific brain regions on the basis of their neuronal activity. Our goal was to examine the effects of SI on behavior changes and to explore the therapeutic potential of DBS in treating social isolation to provide new ideas for finding intervention measures to alleviate physical diseases caused by psychological and social factors.

## 2. Materials and methods

### 2.1. Animals and preparation

Male C57BL/6J mice were acquired from the Soochow University Laboratory Center. The mice were housed in the SPF animal room at the Soochow University Experimental Animal Center with unlimited access to food and water. Mice were randomly divided into socially isolated mice and group-housed mice. All experimental procedures were in accordance with the Animal Research: Reporting of *In Vivo* Experiments guidelines and were approved by the institutional animal care and use committee of Soochow University.

### 2.2. Model establishment

For the adolescence social isolation mouse model, three- or five-week-old mice were individually housed in cages for a period of 1, 6 or 12 months. For the adult social isolation mouse model, eight-week-old mice were individually housed in cages for a period of 2 months. When group-housed, mice were housed five per cage.

### 2.3. Deep brain stimulation

The mPFC of all SI sham and SI DBS mice were implanted with stainless steel microelectrodes (KedouBC, China) (bare electrode diameter, 0.05 mm; length, 3.2 mm; impedance control range, 15–45 kΩ). The procedure for microelectrode implantation was as follows. After being anesthetized, mice were implanted with microelectrodes using a stereotactic instrument. A hole of 2 mm diameter was drilled gently in the skull to implant the microelectrode in the right mPFC with the following parameters: mPFC: +1.98 mm anteroposterior (AP) and − 2.2 mm dorsoventral (DV). After implantation, the microelectrode was sealed with one drop of glue and fixed to the skull using acrylic denture base material. The mice were then returned to their cage and allowed to rest for 7 days. The DBS parameters were set at 130 Hz frequency, 90 μs pulse width, and 100 mA intensity and were applied daily for 60 min during free movement for 14 consecutive days.

### 2.4. Hot-plate test

The hot plate test was used to assess the response of a mouse to heat-induced pain. The test involved placing a mouse on a metal plate that had been heated to 52°C. The reaction time, i.e., the time for the mouse to show signs of experiencing pain, such as licking or lifting a paw, was recorded. After each test, the metal plate was thoroughly cleaned with 30% ethanol to eliminate any residual scent.

### 2.5. Three-chamber test

We employed a white, three-chambered apparatus with opaque acrylic and two interconnecting doors for our experiment. Each subject mouse underwent three testing sessions: (1) habituation: the mouse was placed in the central chamber of a clear, divided Plexiglas box for 20 min to acclimate. (2) Social preference test: following habituation, the subject mouse encountered a never-before-met intruder in one metal cage and an empty cage in the “sociability” session. The time spent in each of the three boxes and in communication with the objects/stranger mouse was recorded within 10 min. (3) Social recognition test: the subject then encountered the first intruder and a second, never-before-met intruder in another pencil cup in the “social novelty” session. The time spent in each of the three boxes and in communication with the two stranger mice, as well as the shuttle time between boxes, were recorded within 10 min.

### 2.6. Resident-intruder test

We utilized a modified protocol in the experiment (33). The subject mouse was placed in a cage with an intruder (a same-sex control mouse of slightly smaller size unfamiliar to the subject) for 10 min. The aggressive behaviors of the resident mouse toward the intruder were recorded, including lateral threats, upright posture, twisting attack, lying down, and chasing, to measure the attack level. Nonaggressive social behaviors such as social exploration, anal/genital sniffing, and social grooming were considered “nonaggressive” and noninteractive in the resident-intruder (RI) test.

### 2.7. Open field test

The mice were individually placed in the center of a 40 × 40 × 40 cm open field and allowed to explore freely for 10 min. During this time, their average speed and the amount of time spent in the center zone were tracked and recorded using ANY-maze software.

### 2.8. Elevated plus maze test

As previously described (34), the elevated plus maze (EPM) apparatus consisted of two open arms, two perpendicular closed arms, and a central platform. The mice were placed facing the closed wall and allowed to explore freely for 5 min. The number of entries into the open arms, the duration of time spent in the open arms, and the proportion of mice entering the open arms were recorded. The results showed that a lower number of entries into the open arms and shorter duration in the open arms indicated higher levels of anxiety-like behavior in the mice.

### 2.9. Tail suspension test

The tail suspension test is a test to measure depression-like behavior in mice. A mouse was suspended by its tail, with the tail being fixed with adhesive tape. The mouse was then hung on a bracket, with its head is 15 cm away from the table. The stationary time during the last 5 min of a 6 min suspension period was recorded with the help of a camera that has a background that is different in color from the fur of the mouse.

### 2.10. Object location test

Before the test, the subject mice were acclimated to the testing arena for 10 min to provide them with a spatial cue. After a 15 to 30 min interval, two identical objects were placed in opposite corners on the same side of the box. The mice were then allowed to explore the objects for 10 min, and the time they spent communicating with each object was recorded. If there was no difference in the preference index between the mice in the experimental group and the control group, the next stage was initiated. Twenty-four hours later, one of the objects was moved to a new location, and the mice were placed in the center of the box floor. The mice were given 5 min to explore the objects, and the time spent communicating with each object was recorded. The recognition index of the mouse was then calculated as (Time spent communicating with the object in the new location)/(Total time spent communicating with both objects) × 100%.

### 2.11. Novel object recognition test

The NOR test protocol was similar to that in a previous study (35). For three consecutive days, each mouse was habituated to the open field environment for 10 min per session. During the training trial, each mouse was placed in an arena containing two identical objects and was given 10 min to explore. Approaching an object was defined as having the nose within 2 cm of it. The preference index of the mouse was then recorded to account for the position of the object. The testing session was conducted 90 min after the training trial. During this session, one of the objects from the training trial was replaced with a novel object, and the mouse was placed back in the arena for 10 min. The time spent actively exploring each object was recorded using the ANY-maze system. The recognition index, which reflects the memory of the mouse for the familiar and novel objects, was calculated as (Time spent exploring the novel object)/(Time spent exploring both objects) × 100%.

### 2.12. Morris water maze test

The Morris water maze (MWM) test is a commonly used method to assess spatial learning and memory in animals. As previously described (36), the apparatus consisted of a metal pool divided into four quadrants, with a platform (8 cm in diameter) placed in one of the quadrants as an escape for the animals. The pool was filled with water to a height of 1.5 cm above the platform, and the water temperature was maintained at 26 ± 1°C. The experiment consisted of two parts: five days of training and a test day on the sixth day. The mice were trained four times a day with intervals of 20 to 30 min. During each training session, the mice were gently placed into the maze facing the wall. On average, the mice found the platform within 90 s and remained on it for 8 s. If the platform was not found within 90 s, the mice were guided to it, and the latency time was recorded as 90 s. After each trial, the mice were dried with a towel and placed in a heated cage. On the sixth day, the platform was removed, and the mice were tested as usual. The results are the time spent in the target quadrant and the latency to find the platform. ANY-maze tracking software was used to record the latency, frequency, and swimming speed of the mice before the platform was found.

### 2.13. Immunofluorescence staining

The mice were anesthetized using 3.6% chloral hydrate (10 mg/kg, intraperitoneal injection). The mice were then perfused with 0.1% phosphate buffer (PB) and 2% paraformaldehyde (PFA) dissolved in 0.1% PB. Brain tissue was carefully removed and placed into a 50 mL EP tube containing 2% PFA for fixation in a refrigerator at 4°C for 2–4 h. Next, 2% PFA was replaced with 15% sucrose, and the sample was kept overnight in the refrigerator at 4°C. Then, 15% sucrose was replaced with 30% sucrose, and the sample was kept overnight in the refrigerator at 4°C. Once the mouse brain tissue sank to the bottom of the centrifuge tube, it was ready for slicing.

For the immunofluorescence staining procedure, the slices were infused with 0.01 M PBS for three 10 min sessions, followed by infusion with 0.5% Triton X-100 in 0.01 M PBS for three 10 min sessions. The slices were then preincubated for 1 h in 10% fetal bovine serum (FBS) before being incubated with primary antibodies overnight at 4°C. After washing the sections in PBS for three 10 min sessions, they were incubated with secondary antibodies for 1 h. The sections were washed again in PBS for three 10 min sessions before being sealed with a sheet containing DAPI. The slices can be stored briefly in the refrigerator at 4°C.

### 2.14. Primary and secondary antibodies

The following antibodies were utilized: Anti-Myelin Basic Protein Mouse (808,401, Biolegend), Anti-APC (Ab-7) Mouse mAb (CC-1) (OP80-100 UG, Merck Millipore), Anti-ASPA/Nur7 (ABN1698, Merck Millipore), Anti-mPDGFRα (AF1062, R&D Systems), Anti-Caspr (55417-1-AP, Proteintech), Anti-c-Fos (9F6) Rabbit mAb (2,250 s Cell Signaling), Alexa Fluor 488-conjugated Donkey anti-Goat IgG (H + L) Cross-Adsorbed Secondary Antibody (A-11055, Invitrogen), Alexa Fluor 555-conjugated Donkey anti-Goat IgG (H + L) Cross-Adsorbed Secondary Antibody (A-21432, Invitrogen), Alexa Fluor 488-conjugated Donkey anti-Mouse IgG (H + L) Highly Cross-Adsorbed Secondary Antibody (A-21202, Invitrogen), Alexa Fluor 555-conjugated Donkey anti-Mouse IgG (H + L) Highly Cross-Adsorbed Secondary Antibody (A-32773, Invitrogen), and Alexa Fluor 488-conjugated Donkey anti-Rabbit IgG (H + L) ReadyProbes^™^ Secondary Antibody (R37118, Thermo Fisher Scientific).

### 2.15. Statistical analysis

All statistical analyses were conducted using SPSS 20.0, and the results are presented as the mean ± standard error of the mean (SEM). Independent sample *t*-tests were used to compare data between two groups, while one-way analysis of variance (ANOVA) followed by Fisher’s LSD *post hoc* tests were used to compare data between multiple groups. Two-way ANOVA was used to analyze data from the Morris water maze (MWM) test. A significance level of *p* < 0.05 was used for all analyses. The symbols ^*^, ^**^, and ^***^ indicate significance levels of *p* < 0.05, *p* < 0.01, and *p* < 0.001, respectively.

## 3. Results

### 3.1. Social isolation starting from adolescence impairs social behaviors in a duration-dependent manner

**Figure 1:**
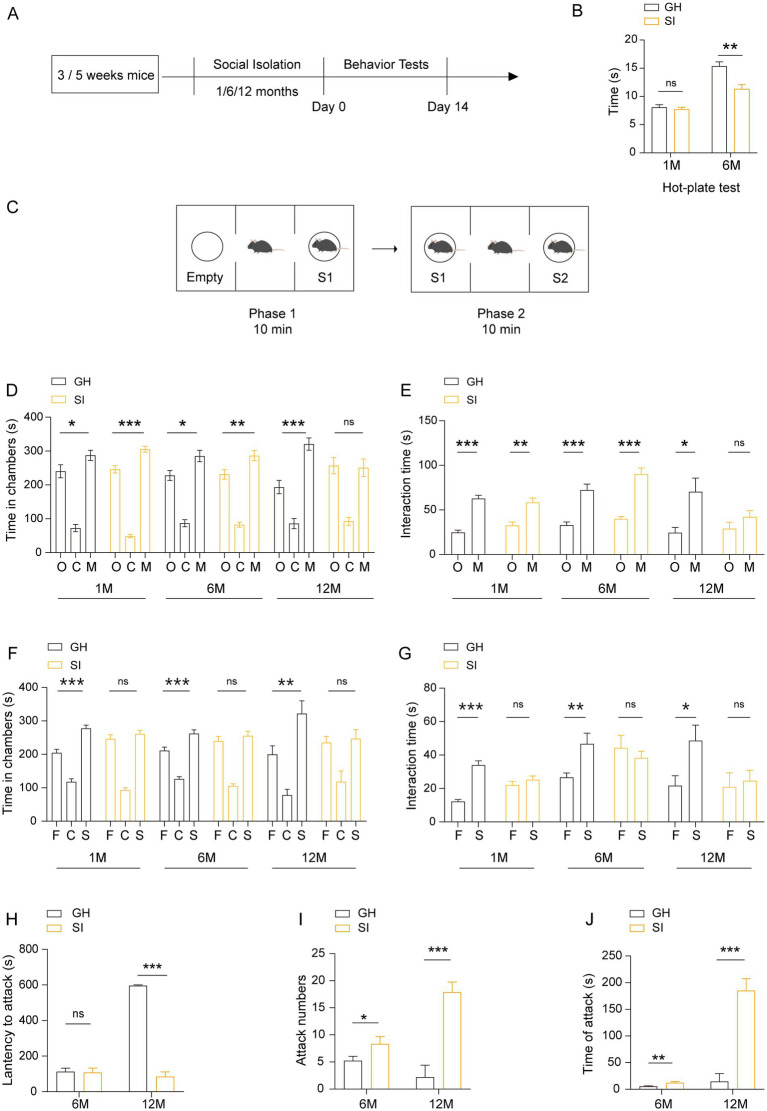
Social isolation of adolescent mice leads to pain hypersensitivity, impaired social capability and aggression. **(A)** Schematic description of the experimental timeline. Three- or five-week-old C57BL/6J male mice were socially isolated for 1, 6 or 12 months. The behavior tests were performed after social isolation. **(B)** Hot-plate test. The latency to onset of pain response in mice in the hot-plate test. **(C–G)** Three-chamber tests. Time that mice spent in chambers and interacting with mice or objects. O: object, C: center, M: mouse, F: familiar, S: stranger. **(H–J)** Resident-intruder test. The latency to start attack **(H)**. Number of attack **(I)**. Total time of attack **(J)**. GH, group housing; SI, social isolation. Data are presented as the mean ± SEM. *n* = 9–13 per group. One-way ANOVA followed by LSD *post hoc* tests **(D,F)**. Two-tailed *t-*test (**E,G,H–J**). ^*^p < 0.05; ^**^p < 0.01; ^***^p < 0.001; ns: nonsignificant.

To investigate the influence of distinct durations of social isolation on adolescent mice, we bred 3 to 5 weeks-old male mice in individual houses for 1, 6 and 12 months. Mice of the same age and sex were bred in a group house as controls (Figure 1A). The mice were subjected to behavioral tests after social isolation. In the hot-plate test, the mice that were socially isolated (SI) for 6 months exhibited a shorter latency to respond to heat-induced pain than group-housed (GH) mice. In contrast, mice of the same age that were socially isolated for 1 month exhibited comparable latency in response to heat-induced pain as GH mice (Figure 1B). These results indicate that long-term social isolation starting (6 months) from adolescence leads to pain hypersensitivity in mice, while short-term social isolation (1 month) is not sufficient to cause alterations in pain sensitivity. In the three-chamber social test, GH mice exhibited normal social novelty preference (Figures 1C–E) and social recognition capability (Figures 1C,E,F), as reflected by the fact that they spent more time in the stranger mouse-containing chamber than in either the empty cage (Figure 1D)-or the familiar mouse-(Figure 1F)-containing chamber and more time interacting with stranger mice than in either the empty cages (Figure 1E) or with familiar mice (Figure 1G) when they were put into an apparatus containing three separated chambers where one stranger mouse was in one side chamber, while another side chamber contained either an empty cage (Figures 1D,E) or a familiar mouse (Figures 1F,G). The mice reared under social isolation for either 1 month or 6 months spent more time in the stranger mouse-containing chambers than in the empty cage-containing chambers (Figure 1D) and more time interacting with stranger mice than in the empty cages (Figure 1E), indicating that both 1 month and 6 months durations of social isolation starting from adolescence do not affect the social novelty preference of mice. In contrast, the ability to recognize stranger mice was lost when they were exposed to familiar mice and stranger mice, as they spent more time staying in the stranger mouse-containing chambers (Figure 1F) and interacting with stranger mice (Figure 1G). The results indicate that both 1 month and 6 months durations of social isolation starting from adolescence impair the social recognition capability of mice. Notably, mice socially isolated for 12 months failed to show a preference for stranger mice when they were challenged by either empty cages or familiar mice versus stranger mice (Figures 1D–G), indicating that 12 months of social isolation impairs both the social preference and social recognition capacity of mice. These results indicate that distinct durations of social isolation result in social capability impairment to different extents. Short-term social isolation starting in adolescence impairs social memory, while long-term social isolation starting in adolescence impaired social ability and social memory in mice. In the resident-intruder test, mice socially isolated for either 6 months or 12 months showed increased numbers (Figure 1I) and durations (Figure 1J) of attacking intruder mice. Twelve months-old socially isolated mice exhibited a shorter latency to attack intruder mice than 6 months-old socially isolated mice (Figure 1H). These results indicate that long-term social isolation starting in adolescence enhances aggression in mice. In summary, these results indicate that social isolation starting in adolescence impairs social behaviors in a duration-dependent manner. Short-term social isolation is sufficient to impair social recognition capability, whereas long-term social isolation impaired social capability, including social novelty preference and social recognition capability, leading to enhanced aggression.

### 3.2. Social isolation starting in adolescence increases spontaneous locomotor activity and anxiety-like and depressive-like behaviors in mice in a duration-dependent manner

**Figure 2:**
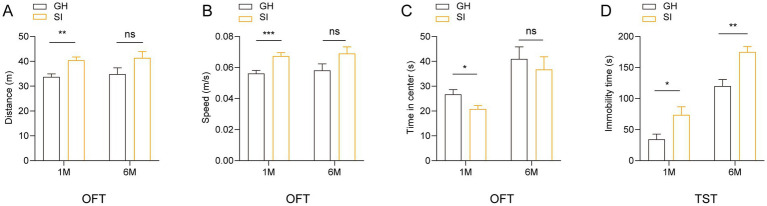
Social isolation of adolescent mice leads to anxiety-like and depression-like behaviors. Three- or five-week-old C57BL/6J male mice socially isolated for 1 or 6 months were subjected to behavioral tests. **(A–C)** Open field test (OFT). Total distances. **(A)** Mean speed. **(B)** Time mice spent in the center field **(C)**. **(D)** Tail suspension test (TST). Time of immobility. Data are presented as the mean ± SEM. *n* = 22–25 per group in 1 month social isolation. *n* = 11–14 per group in 6 months social isolation. Two-tailed *t*-test **(A–D)**. ^*^p < 0.05; ^**^p < 0.01; ^***^p < 0.001; ns: nonsignificant.

Social isolation stress, as a kind of chronic psychiatric stress, may induce the changes of emotional behavior; thus, we examined the effects of distinct durations of social isolation on emotions. We observed that 3 to 5 week-old mice under social isolation for 1 month exhibited increased running distances (Figure 2A) and enhanced running velocity (Figure 2B) in the open field test (OFT) compared to GH mice, indicating that 1 month of social isolation during adolescence results in hyperactivity of spontaneous locomotion in mice. Notably, adolescent mice under social isolation for 1 month spent less time in the center area than GH mice (Figure 2C), indicating that 1 month of social isolation during adolescence increases the anxious behavior of mice. In contrast, mice under social isolation for 6 months failed to exhibit significant differences in the OFT compared with GH mice (Figures 2A–C), suggesting that long-term isolation fails to affect the spontaneous activity and anxious behavior of mice. In the tail suspension test (TST), compared to GH mice, mice under social isolation for both 1 month and 6 months spent more time staying immobile (Figure 2D), indicating that both short-term and long-term durations of social isolation increase the depressive behavior of mice.

### 3.3. Social isolation starting in adolescence results in spatial memory impairment accompanied by a decline in willingness to learn

**Figure 3:**
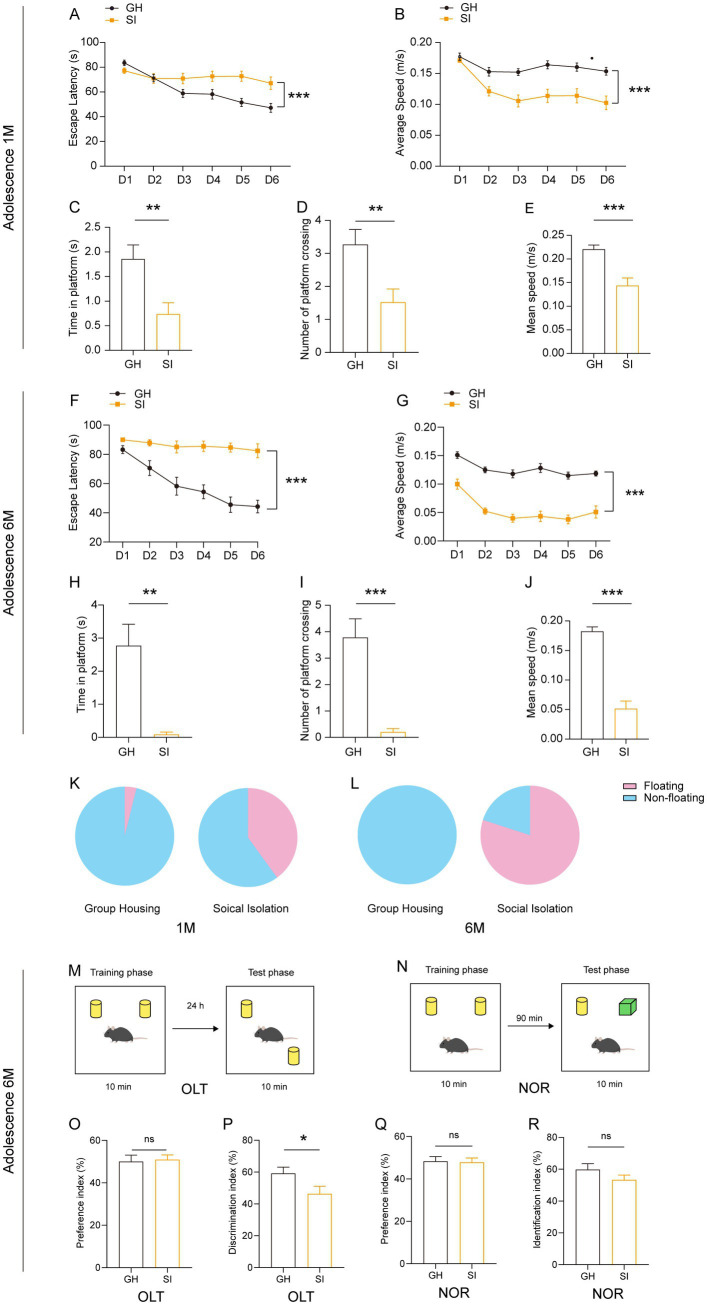
Social isolation of adolescent mice leads to a decreased willingness to learn and impaired short-term spatial memory. Three- or five-week-old C57BL/6J male mice socially isolated for 1 or 6 months were subjected to behavioral tests. **(A–E)** Analysis of adolescent mouse behaviors after 1 month of social isolation in the Morris water maze (MWM). The escape latency **(A)** and average speed **(B)** to find the platform in the learning phase. The escape latency **(C)**, number of platform crossings **(D)** and speed **(E)** in the probe test. **(F–J)** Analysis of adolescent mouse behaviors after 6 months of social isolation in the MWM. The escape latency **(F)** and average speed **(G)** to find the platform in the learning phase. The escape latency **(H)**, number of platform crossings **(I)** and speed **(J)** in the probe test. **(K,L)** The floating proportion of 1 month and 6 months SI mice in the MWM probe test. The pink part represents floating mice, and the blue part represents nonfloating mice. **(M–R)** Analysis of short-term memory in adolescent mouse behaviors after 6 months of social isolation. **(M,O,P)** Object location task (OLT). Preference index **(O)**. Discrimination index **(P)**. **(N,Q,R)** Novel object recognition test (NOR). Preference index **(Q)**. Identification index **(R)**. The data shown represent the mean ± SEM. *n* = 22–25 per group in 1 month social isolation. *n* = 11–14 per group in 6 month social isolation. Two-tailed *t*-test **(C–E,H–J,O–R)**. Two-way ANOVA followed by LSD *post hoc* test **(A,B,F,G)**. ^*^p < 0.05; ^**^p < 0.01; ^***^p < 0.001; ns: nonsignificant.

We further examined the influence of social isolation on learning and memory in mice. We observed that in the Morris water maze (MWM) test, adolescent mice under social isolation for either 1 month or 6 months exhibited increased escape latency in the learning period (Figures 3A,F) and decreased time spent at the spot where the platform was originally located and numbers of crossings of the spot in the probe trial compared with GH mice (Figures 3C,D,H,I). These SI mice also showed a slower swimming speed in the learning period as well as the probe trial than GH mice (Figures 3B,G,E,J). The higher proportion of socially isolated mice floating on the water rather than swimming indicates a decline in willingness to learn (Figures 3K,L). To further examine the influences of social isolation on learning and memory, we performed an object location test (OLT) (Figure 3M) and novel object recognition (NOR) test (Figure 3N). In the OLT, adolescent mice under social isolation for 6 months exhibited a decreased discrimination index compared to GH mice (Figure 3P). However, both SI and GH mice exhibited comparable preference indices (Figure 3O), indicating that the decreased discrimination index in SI mice was not caused by a location preference. These results indicate that long-term social isolation starting in adolescence impairs the spatial memory of mice. In contrast, SI mice showed a recognition index comparable to that of GH mice in the NOR test, which is designed for testing nonspatial memory (37) (Figures 3Q,R), indicating that social isolation does not impair nonspatial memory.

### 3.4. Social isolation during adulthood leads to locomotor hyperactivity and the impairment of social recognition ability and fear memory

**Figure 4:**
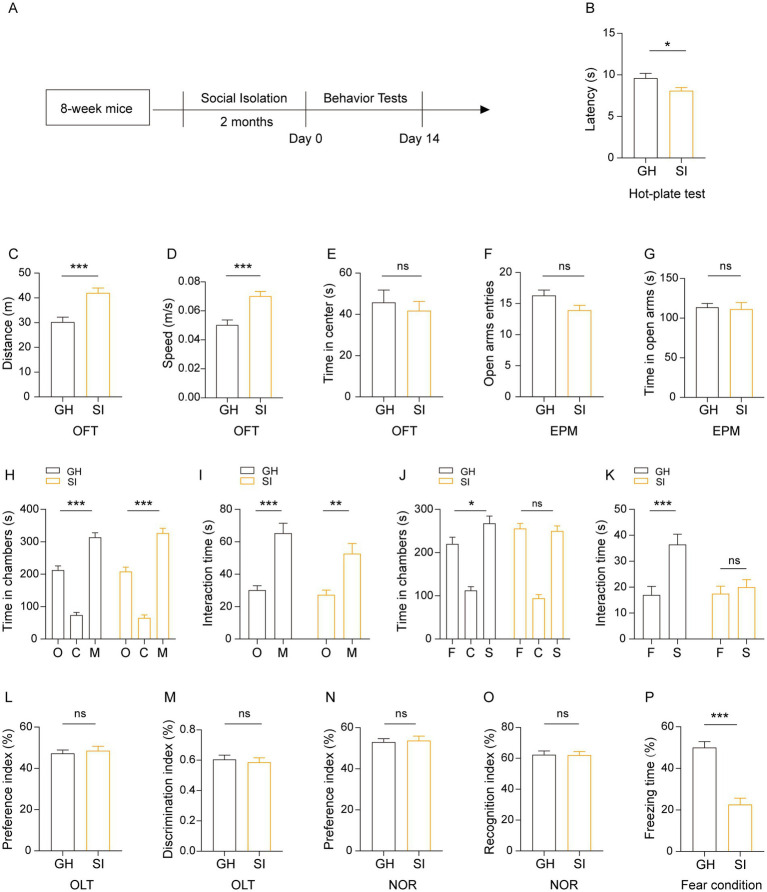
Social isolation of adult mice leads to pain hypersensitivity, impaired social recognition memory and a disturbance of fear memory formation. **(A)** Schematic description of the experimental timeline. Eight-week-old C57BL/6J male mice were socially isolated for 2 months. The behavior tests were performed after social isolation. **(B)** Hot-plate test. The latency to onset of pain response in mice in the hot-plate test. **(C–E)** Open field test (OFT). Total distances **(C)**. Mean speed **(D)**. Time mice spent in the center field **(E)**. **(F,G)** Elevated plus maze (EPM). Entries in the open arms **(F)**. Time spent in the open arms **(G)**. **(H–K)** Three-chamber tests. Time that mice spent in chambers **(H,J)** and interacting with mice or objects **(I,K)**. O: object, C: center, M: mouse, F: familiar, S: stranger. **(L,M)** Object location task (OLT). Preference index (L). Discrimination index **(M) (N,O)** Novel object recognition (NOR) test. Preference index **(L)**. Identification index **(O)**. **(P)** Contextual fear conditioning. The proportion of freezing time in the test phase. The data shown represent the mean ± SEM. *n* = 14–15 per group. One-way ANOVA followed by LSD *post hoc* test **(H,J)**. Two-tailed *t*-test **(B–G,I,K,L–P)**. ^*^p < 0.05; ^**^p < 0.01; ^***^p < 0.001; ns: nonsignificant.

We further examined how social isolation during adulthood affected the behaviors of mice. Eight-week-old male mice were socially isolated for 2 months by being housed alone. Age- and sex-matched mice that were group housed were used as controls (Figure 4A). In the hot-plate test, SI mice showed a decreased latency in response to heat compared to GH mice (Figure 4B), indicating that social isolation during adulthood causes pain hypersensitivity in mice. Similar to mice that were socially isolated starting in adolescence for 1 month (Figure 2A), mice that were socially isolated during adulthood exhibited increased running distances (Figure 4C) and running velocity (Figure 4D) in the OFT, indicating that social isolation during adulthood results in hyperactivity of spontaneous locomotor activity. Compared with GH mice, SI mice exhibited comparable levels of time spent in the center area in the OFT (Figure 4E) as well as numbers of entries into and the time spent in the open arms in the elevated plus maze (EPM) test (Figures 4F,G), indicating that social isolation during adulthood does not increase anxious behavior in mice. In the three-chamber social test, SI mice exhibited a preference for stranger mice versus empty cages, as reflected by the fact that they spent more time staying in the side chamber containing stranger mice than in the side chamber containing empty cages (Figure 4H) and interacting with stranger mice than in the side chamber containing empty mice (Figure 4I). In contrast, SI mice failed to exhibit a preference for stranger mice versus familiar mice, as evidenced by the comparable time spent either staying in chambers containing stranger and familiar mice (Figure 4J) or interacting with stranger and familiar mice (Figure 4K). These results indicate that social isolation during adulthood causes an impairment in social recognition ability while the social preference of mice is preserved. We also examined the effects of such a social isolation scheme on the cognition of mice. The results revealed that SI mice exhibited similar performance in both the OLT (Figures 4L,M) and NOR test (Figures 4N,O). In contrast, SI mice exhibited decreased freezing time in contextual fear conditions compared to GH mice (Figure 4P). These results indicate that social isolation during adulthood impairs fear memory but does not influence spatial or episodic memory.

### 3.5. Social isolation results in hypomyelination in various brain regions

**Figure 5:**
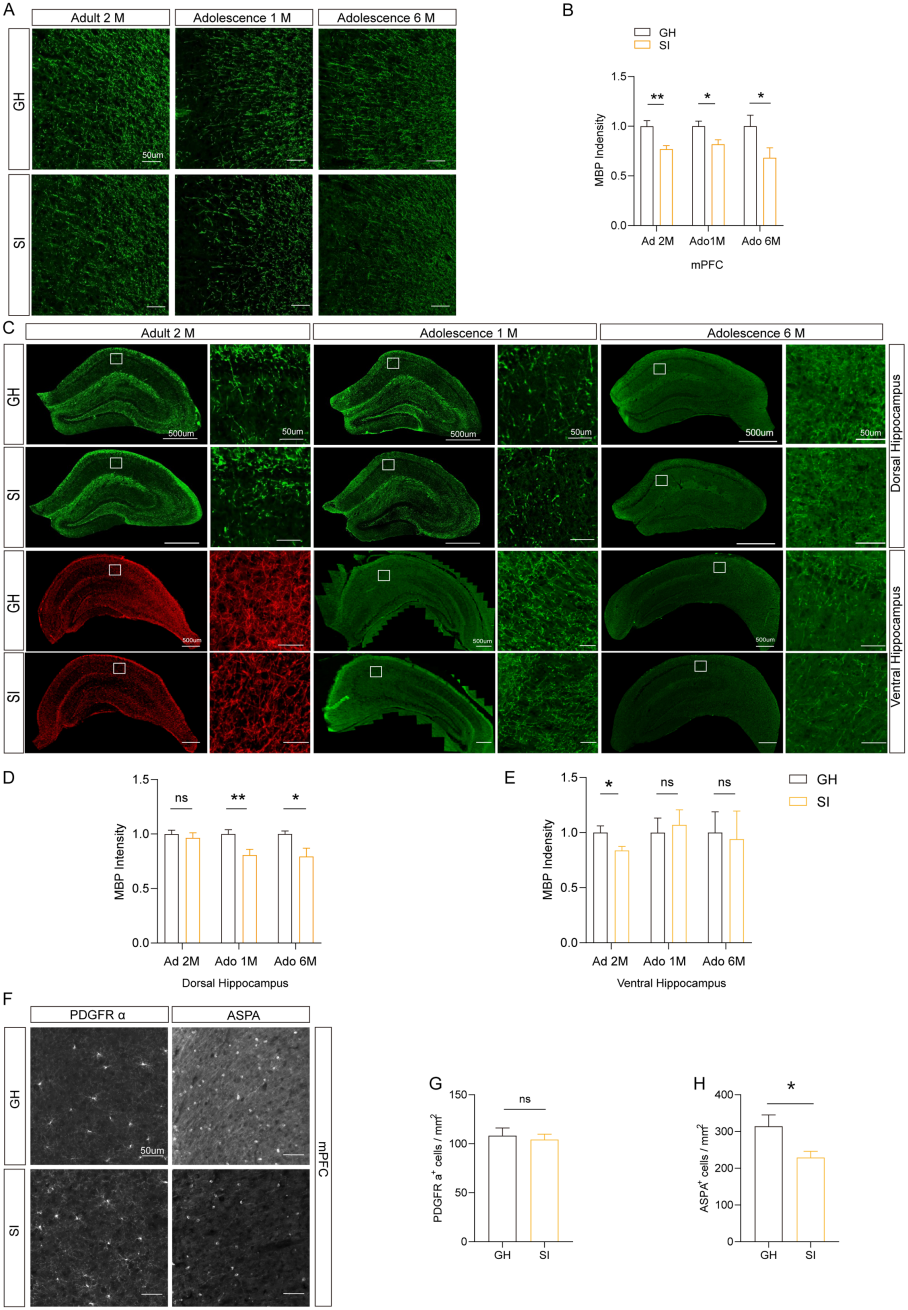
Different durations of social isolation decrease myelin in the mPFC. **(A,B)** Immunostaining of MBP in the medial prefrontal cortex (mPFC). The intensity of MBP staining was quantified. **(C–E)** Analysis of the intensity of MBP staining in the dorsal hippocampus and ventral hippocampus by immunostaining. **(F)** Representative images of different markers. **(G)** Analysis of the number of PDGFR α^+^ cells in the mPFC. **(H)** Analysis of the number of ASPA^+^ cells in the mPFC. Ad: adult; Ado: adolescence. The data shown represent the mean ± SEM. *n* = 17–33 from 3 mice per group in 2 months social isolation of adult mice. *n* = 9–10 from 3 mice per group in 1 month social isolation of adolescence. *n* = 10–15 from 3 mice per group in 6 months social isolation of adolescence. Two-tailed *t*-test **(B,D,E,G,H)**. ^*^p < 0.05; ^**^p < 0.01; ns: nonsignificant. Scale bars: 50 μm **(A,F)**. 500 μm in images with lower magnification, 50 μm in images with higher magnification **(C)**.

Previous research has shown that social isolation during adolescence and adulthood induces deficits of myelination without causing other obvious ultrastructural changes (11, 12). However, how the content of myelin in the brain regions changes with distinct durations and starting age stages of social isolation is unclear. Therefore, we investigated the effect of social isolation on myelin formation by analyzing the content of myelin basic protein (MBP), which exhibits reduction in response to social isolation (social isolation impairs remyelination in mice through modulation of IL-6; clemastine enhances myelination in the prefrontal cortex and rescues behavioral changes in socially isolated mice). We focused on three brain regions, the medial prefrontal cortex (mPFC) and ventral and dorsal hippocampus, which are linked to social behaviors, memory, and emotion. Our results revealed that compared to that in GH mice, the MBP intensity was reduced in the mPFC of SI mice with distinct social isolation schemes, including social isolation for 2 months during adulthood and social isolation for either 1 month or 6 months starting from adolescence. Despite the differences in behaviors, the change in MBP staining intensity in distinct mouse models of SI was similar (Figures 5A,B). In contrast, decreased MBP staining intensity in the dorsal hippocampus was observed in mice under social isolation starting from adolescence for either 1 month or 6 months but not in mice under social isolation for 2 months during adulthood (Figures 5C,E). Interestingly, decreased MBP staining intensity in the ventral hippocampus was observed only in SI mice under social isolation during adulthood but not in mice socially isolated starting from adolescence for either 1 month or 6 months (Figures 5C,D). Therefore, distinct schemes of social isolation result in the occurrence of hypomyelination in different brain regions. In the mPFC of mice under 12 months of social isolation, we observed that the numbers of ASPA^+^ oligodendrocytes (OLs), which represent myelinating OLs (38, 39), were decreased compared to those in GH mice (Figures 5F,H). However, the density of PDGFRα^+^ oligodendrocyte precursor cells (OPCs) remained identical in the mPFC of SI and GH mice (Figures 5F,G). These results suggest that social isolation impairs myelin content by reducing the number of myelinating OLs.

### 3.6. Cell activity decreases in the mPFC and dorsal hippocampus of mice experiencing social isolation

**Figure 6:**
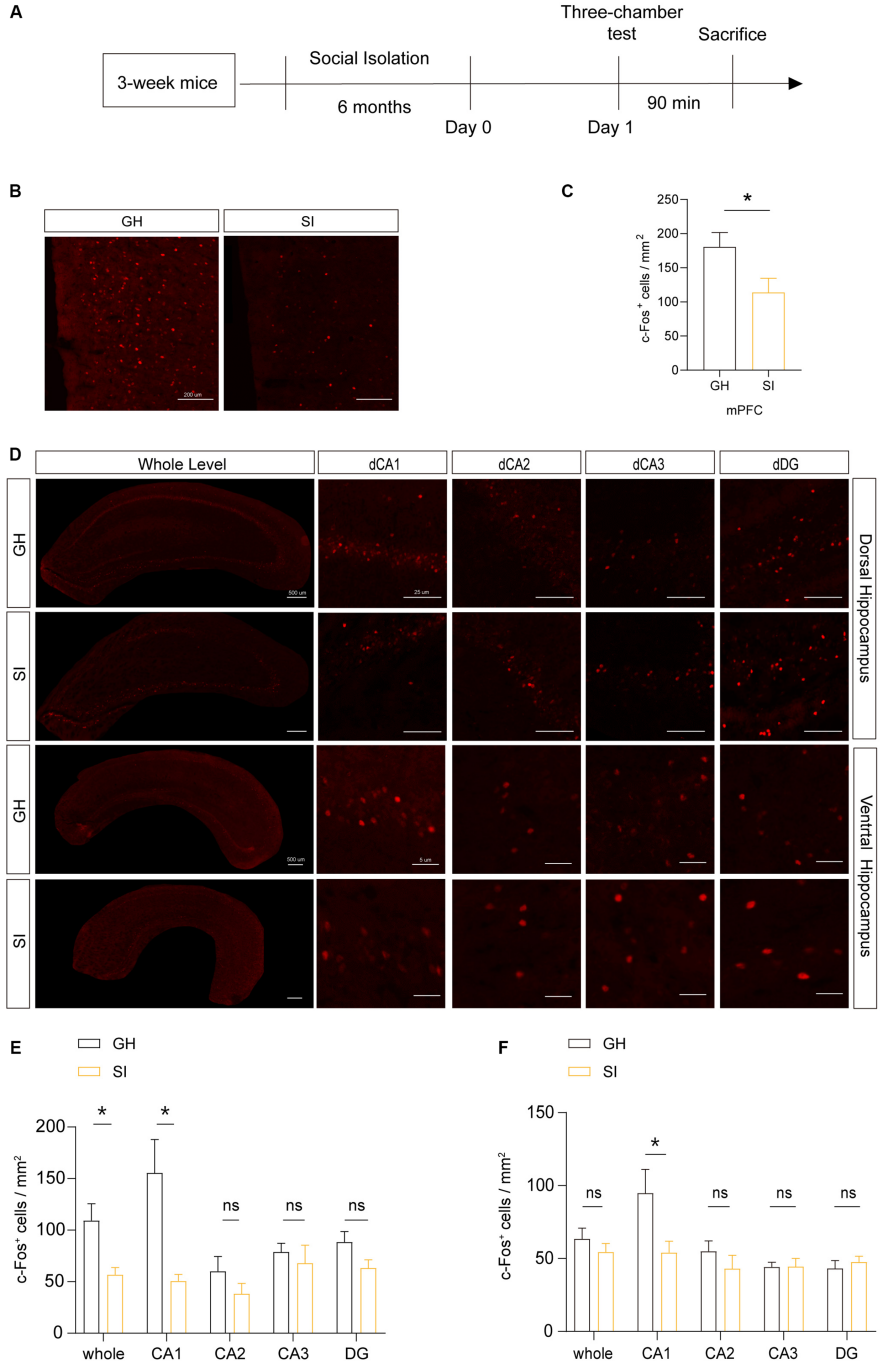
Social isolation of adolescent mice influences the activity of neurons in the mPFC after social interaction. **(A)** Schematic description of the experimental timeline. Three-week-old C57BL/6J male mice were socially isolated for 6 months. Mice were sacrificed 90 min after the three-chamber test to detect the number of active neurons by immunostaining for c-Fos. **(B–F)** Analysis of the number of c-Fos^+^ cells in the mPFC, dorsal hippocampus and ventral hippocampus by immunostaining. Numbers of c-Fos^+^ cells per square millimeter. The data shown represent the mean ± SEM. *n* = 10–15 from 3 mice per group in 6 months social isolation of adolescence. Two-tailed *t*-test **(C,E,F)**. ^*^p < 0.05; ns: nonsignificant. Scale bars: 200 μm in images of the mPFC **(B)**. 500 μm at lower magnification and 50 μm at higher magnification in images of the dorsal and ventral hippocampus **(D)**.

Because normal conduction of nerve impulses relies on the insulating properties of myelin around axons, the change in myelin content may influence the activity of neurons. Additionally, distinct types of cells in the brain have functionally and structurally interactive connections, so the activity of different cells can influence each other. To investigate whether the activity of cells will change under the stimulation of social interaction in distinct brain areas of mice experiencing social isolation, we examined the number of c-Fos^+^ cells in the brain areas of SI mice after social stimulation. Ninety minutes after being exposed to stranger mice in the three-chamber social test, mice were sacrificed for c-Fos immunostaining. The results showed that c-Fos^+^ cells were decreased in the mPFC and dorsal hippocampus of SI mice compared to those in GH mice (Figures 6A–E). Notably, the number of c-Fos^+^ cells in the dorsal CA1 region, but not the dorsal CA2 or CA3 region, was significantly decreased in SI mice compared to GH mice (Figures 6D,E). Although the average density of c-Fos^+^ cells remained identical in the ventral hippocampus between SI and GH mice, c-Fos^+^ cells showed a decreased density in ventral CA1 as well (Figures 6D,F). These results indicate that the activity of cells in response to social stimulation in the mPFC and ventral and dorsal CA1 regions was suppressed by social isolation.

### 3.7. DBS leads to a recovery of the social-isolation-impaired social novelty preference

**Figure 7:**
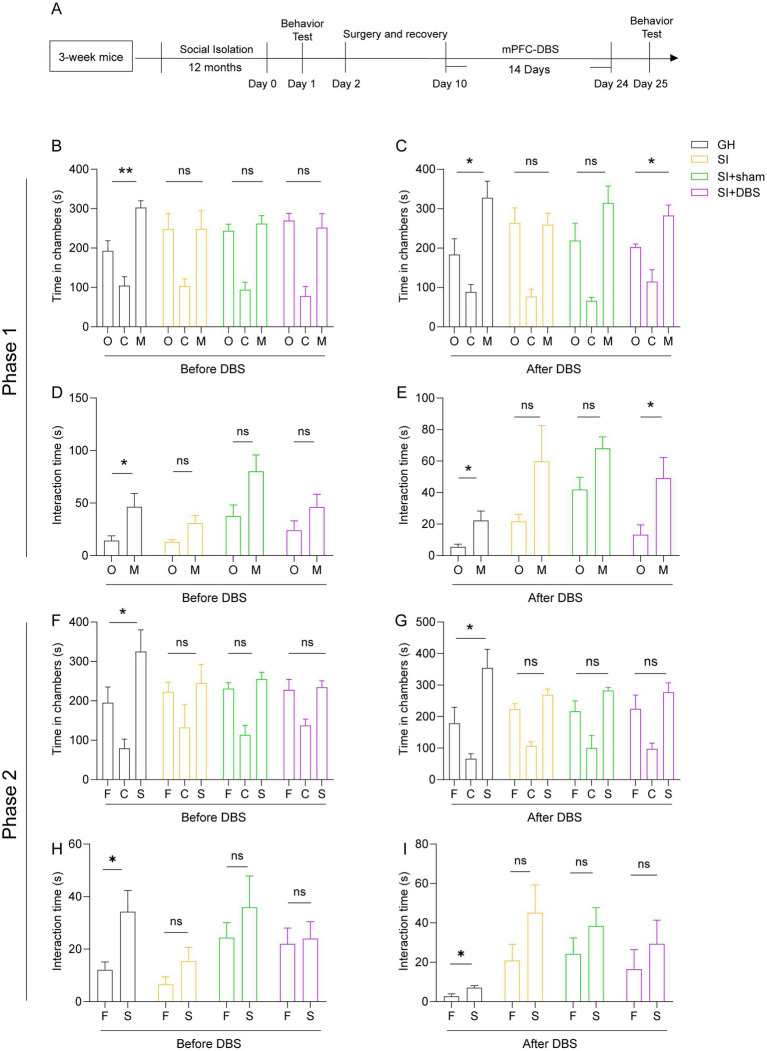
DBS restores the impaired social ability caused by 12 months of social isolation from adolescence but not the impaired social recognition memory. **(A)** Schematic description of the experimental timeline. Three-week-old C57BL/6J male mice were socially isolated for 12 months. DBS was performed for 14 days after social isolation. The behavior test was performed before and after DBS. **(B–I)** Three-chamber test. Time that mice spent in chambers **(B,F)** and interacting with mice or objects **(D,H)** before DBS. Time that mice spent in chambers **(C,G)** and interacting with mice or objects **(E,I)** after DBS. The data shown represent the mean ± SEM. *n* = 5 per group. One-way ANOVA followed by LSD *post hoc* tests **(B,C,F,G)**. Two-tailed *t*-test **(D,E,H,I)**. ^*^p < 0.05; ^**^p < 0.01; ns: nonsignificant.

DBS exhibits great therapeutic potential in various neurological disorders, such as PD, AD, dementia and Rett syndrome. We thus examined the therapeutic potential of DBS in mice that were socially isolated for 12 months starting from adolescence. Considering that both hypomyelination and cellular activity in response to social stimulation decrease in the mPFC region, we stimulated this region with DBS for 14 consecutive days. The sham mice were subjected to the same procedures but received no actual stimulation (Figure 7A). As mentioned, such extreme long-term social isolation impairs both the social preference and social recognition ability of mice, as evidenced by the decreased time that SI mice spent staying in the stranger mouse-containing chambers and interacting with stranger mice compared with that of GH mice (Figures 7B–I). The social preference of SI mice that received DBS recovered, as reflected by the increased time that they spent in stranger mouse-containing chambers and interacting with stranger mice versus empty cages compared to SI-sham mice (Figures 7C,E). In contrast, stimulation of the mPFC with DBS did not lead to the recovery of the social recognition ability of SI mice when the mice were challenged by either familiar mice or stranger mice in three-chamber social tests (Figures 7G,I). Thus, stimulation of the mPFC with DBS leads to a recovery of social preference but not social recognition ability, which is impaired by long-term social isolation.

### 3.8. DBS rescues the suppressed cellular activity and OPCs lineage numbers decline caused by social isolation

**Figure 8:**
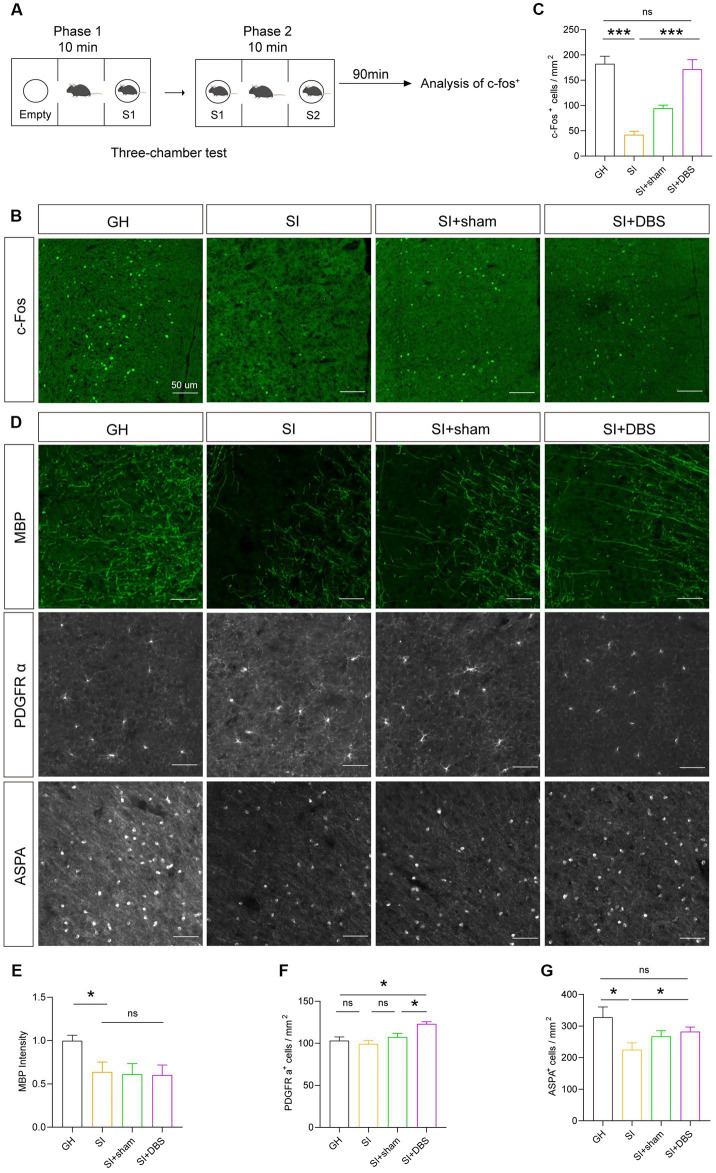
DBS restores the activity of neurons and the number of OPCs and OLs but has no influence on myelin. **(A)** Schematic description of the experimental timeline. **(B)** Representative images of c-Fos^+^ cells in the mPFC. **(C)** Analysis of the number of c-Fos^+^ cells in the mPFC by immunostaining. Numbers of c-Fos^+^ cells per square millimeter. **(D)** Representative images of different markers. **(E)** Immunostaining of MBP in the mPFC. The intensity of MBP staining was quantified. **(F)** Quantification of PDGFR α^+^ cells in the mPFC by immunostaining. Numbers of PDGFR α^+^ cells per square millimeter. **(G)** Analysis of the number of ASPA^+^ cells in the mPFC by immunostaining. Numbers of ASPA^+^ cells per square millimeter. The data shown represent the mean ± SEM. *n* = 10 from 3 mice per group **(B,C)**. *n* = 17–26 from 3 mice per group **(D)**. *n* = 14–22 from 3 mice per group **(E)**. One-way ANOVA followed by LSD *post hoc* tests **(C,E,F,G)**. ^*^p < 0.05; ^***^p < 0.001; ns: nonsignificant. Scale bars: 50 μm **(B,D)**.

Previous research has reported that DBS can increase neuronal activity in many disorders (40, 41). To investigate whether DBS reverse SI-induced neural activity decline, we observed the change in cellular activity by stimulating the mPFC with DBS, as evidenced by the density of c-Fos^+^ cells after social stimulation (Figures 8A–C). SI mice that received DBS stimulation showed an increased density of c-Fos^+^ cells in the mPFC compared to both SI mice and SI-sham mice (Figures 8B,C), indicating that DBS rescued cellular activity decline in response to social stimulation. Although the impaired social preference and suppressed cellular activity were restored by DBS treatment, the hypomyelination, as shown by decreased MBP^+^ intensity in the mPFC of SI mice, was not changed by DBS (Figures 8D,E). Interestingly, the densities of both OPCs (Figures 8D,F) and myelinating OLs (Figures 8D,G) were increased in SI mice that received DBS compared to SI-sham mice. These results indicate that although without rescuing hypomyelination, DBS increases the numbers of OPCs and myelinating OLs.

## 4. Discussion

Social isolation causes various psychological and cognitive problems (42–44). However, it remains unclear how the duration of social isolation affects cognition and emotion. In this article, we described the effects of social isolation of distinct durations during both adulthood and adolescence on the emotion and cognition of mice. Social preference and social recognition ability are the basic elements of social skills. Social preference reflects the social instinct of individuals, while social recognition ability reflects social memory. We herein described that in contrast to social novelty preference, social recognition ability was more vulnerable to social isolation, suggesting that social isolation first impairs social memory rather than social instinct. We observed that social preference was impaired by an extremely long duration of social isolation, indicating that long-term social isolation impairs social instinct. Consistent with this observation, long-term social isolation also causes aggressive behaviors in mice. In addition to social memory, we also observed an impairment in spatial learning due to social isolation. In the MWM test, both short-term and long-term social isolation caused a decline in the willingness of mice to learn. However, in the OLT, in which mice spend less effort learning, adult mice subjected to short-term social isolation failed to show impaired spatial memory. These results indicate that short-term social isolation is more prone to reduce learning willingness rather than impairing learning ability. However, 6 months of social isolation impaired spatial learning and memory in the OLT. Thus, these results reveal that social isolation results in a reduction in learning willingness and then a disruption of spatial learning ability with an increasing duration of social isolation. Notably, fear memory, which is not involved in triggering learning, was impaired by short-term social isolation in adult mice. Given that short-term social isolation during adulthood caused hypersensitivity to pain, the reduced freezing behaviors observed in the fear condition test were not due to an impairment of sensory function. Short-term social isolation resulted in an increase in anxious and depressive behaviors, while long-term socially isolated mice failed to exhibit obvious anxious behaviors. These results indicate that the increase in anxious behaviors may be an intermediate symptom during social isolation in mice. In summary, these results suggest that distinct durations of social isolation may cause different psychological and cognitive problems. Social isolation can manifest in various scenarios, such as during epidemic outbreaks of infectious diseases, within intensive care units, and among the aging population. Findings from animal experiments on the effects of social isolation can enable researchers to intervene earlier, thereby preventing the emergence of negative consequences.

We further analyzed brain regions where cellular activity may be influenced by social isolation. We observed that cellular activity in the mPFC and dorsal and ventral CA1 region decreased in response to social stimulation. This is consistent with the fact that all three regions encode social memory. The dorsal CA1 region is important for the formation of short-term social memory (the temporal binding function of the dorsal CA1 region is critical for declarative memory formation), whereas the ventral CA1 region is crucial for social memory storage (45). A population of pyramidal neurons in the mPFC is responsible for the formation and storage of social memory (46). Thus, these results indicate that social isolation suppresses the activation of neurons in response to social stimulation in broad brain regions involved in social memory. In the past, the c-Fos gene and the c-Fos protein were considered markers of neuronal activity (47), while now they are considered markers of cellular activity (for both neurons and glia) that are easily expressed after a wide range of stimuli (both harmful and nonharmful) with the deepening of research (48). In our research, the number of c-Fos^+^ cells was decreased in the brain areas of SI mice, while the specific type of active cells was not investigated. In future research, other markers of neurons and glia can be used with c-Fos to confirm specific types of cells that are truly reactive to social isolation.

Myelin, as a membrane encasing neuronal axons, plays a crucial role in transmitting nerve signals. Myelin plasticity is required for the formation and consolidation of memory (49). Impaired myelin structure or myelin plasticity is involved in several neurological disorders, such as schizophrenia (50, 51), Alzheimer’s disease (52–54), and frontotemporal dementia (55, 56). Previous studies have shown that social isolation during either adolescence or adulthood leads to a decrease in myelin-associated protein and structural alterations in myelin sheaths in the mPFC region of mice, the latter of which results in reduced social behavior (11, 12), emphasizing a role of myelination in social behaviors. Consistent with these observations, we observed decreased myelin content, as reflected by decreased MBP intensity, in the mPFC of mice socially isolated for distinct durations. However, despite the differences in behaviors, MBP staining intensity was reduced to a similar extent in the mPFC of mice socially isolated for distinct durations. Notably, the MBP staining intensity of other brain areas exhibited different alterations in mice socially isolated during adulthood and starting from adolescence. SI mice isolated for 2 months during adulthood exhibited decreased MBP staining intensity in the ventral hippocampus, whereas mice socially isolated starting from adolescence for a longer time exhibited decreased MBP staining intensity in the dorsal hippocampus. In addition to social memory, the dorsal hippocampus also encodes spatial memory and declarative memory (57, 58). However, the ventral hippocampus is more involved in stress and emotion (59–61). This is consistent with our observation that social isolation starting in adolescence impaired the spatial memory of mice. Consistent with the finding that social isolation impaired the differentiation and maturation of OLs (15, 62), we observed that the density of myelinating OLs rather than that of OPCs was decreased in SI mice. These results suggest that social isolation impairs myelin content by reducing the number of myelinating OLs. Through magnetic resonance imaging (MRI) studies, M I Schubert et al. reported that the volume of the mPFC was reduced in rats experiencing social isolation (63). Some clinical researchers have used MRI to investigate the changes in structure and function in the brains of children who were exposed to social isolation in institutions during early childhood (64, 65). The results showed that brain structure abnormalities, including consistently reduced total gray and white matter volumes, occurred in the prefrontal cortex (PFC) and hippocampus of children who experienced social isolation. Our results support this finding in humans experiencing social isolation. We investigated only some brain areas associated with social memory and social interaction, and other brain regions related to other behaviors can be studied in the future.

DBS is a therapeutic approach that delivers electrical current directly to specific brain regions, potentially modulating pathological activity of various brain circuits. Previous studies have shown that DBS acts on relevant circuit nodes to mitigate cognitive impairment by changing the local field potentials (LFPs) and promoting the release of neurotransmitters in certain regions (40, 41, 66). These actions suggest that DBS could be used to attenuate cognitive impairment in neurological diseases such as AD. In addition to acting on neurons, DBS has also been shown to alter glial function and thus play a regulatory role (67). We examined the therapeutic potential of DBS in alleviating the abnormal behaviors caused by long-term social isolation. Our study showed that the stimulation of the mPFC with DBS restored the impaired social preference, but not the impaired social recognition ability, caused by long-term social isolation in adolescent mice. Consistent with these results, DBS also restored the decreased cellular activity in the mPFC in response to social stimulation. In contrast, the decreased myelin density and myelinating OLs in the mPFC of SI mice failed to be restored by DBS. However, DBS increased the density of OPCs. These results indicate the therapeutic potential of stimulating the mPFC with DBS in individuals with social preference deficits caused by long-term social isolation, as well as the effects of DBS on the cellular activity and density of OPCs. Presently, the link between improvements in behaviors and increased cellular activity after the use of DBS is unclear, and chemogenetics and optogenetics, as good approaches to regulate specific types of cells, can be used to investigate the link in future research.

Although our findings demonstrated that social isolation had negative effects and DBS had therapeutic effects on impaired social preference caused by social isolation, the current study also has some limitations. First, our study used an extreme isolation environment to investigate the effect of social isolation on mice, which cannot truly simulate real-life isolation environments. In future studies, the rearing environment of socially isolated mice will be improved by adding social signals such as visual, olfactory, and auditory cues to better simulate real-life isolation environments. This will help to investigate the effects and underlying mechanisms of social isolation. Second, we found negative effects on behavior and the density of cells in the brain caused by social isolation, but more studies are needed to investigate the specific mechanisms underlying these changes.

## Data availability statement

The original contributions presented in the study are included in the article/supplementary material, further inquiries can be directed to the corresponding authors.

## Ethics statement

The animal study was reviewed and approved by the Institutional Animal Care and Use Committee of Soochow University.

## Author contributions

Y-YH and Q-HM conceived and designed the study. Y-YH and X-SD contributed to data acquisition. GY and X-SL performed statistical analyses. Y-YH and Y-YS contributed to the initial draft of the manuscript and preparation of the figures. LF, RC, and Q-HM contributed to literature research and manuscript preparation. Y-YS, RC, and Q-HM supervised the study. All authors contributed to the article and approved the submitted version.

## Funding

This study was funded by the STI2030-Major Projects (2021ZD0204001), National Natural Science Foundation of China (92049120, 81870897, 81770085, 82070095, and 81901296), the Guangdong Key Project in the Development of New Tools for the Diagnosis and Treatment of Autism (2018B030335001), the Natural Science Foundation of Jiangsu Province (BK20181436), the National Major Scientific and Technological Special Project for Significant New Drugs Development (2019ZX09301102), the Discipline Construction Program of the Second Affiliated Hospital of Soochow University (XKTJ-TD202003), Sino German cooperation and exchange project (M-0679), and the Suzhou Science and Technology Project (SKY2022161).

## Conflict of interest

The authors declare that the research was conducted in the absence of any commercial or financial relationships that could be construed as a potential conflict of interest.

## Publisher’s note

All claims expressed in this article are solely those of the authors and do not necessarily represent those of their affiliated organizations, or those of the publisher, the editors and the reviewers. Any product that may be evaluated in this article, or claim that may be made by its manufacturer, is not guaranteed or endorsed by the publisher.
